# Molecular identification and eco-friendly management of rice brown planthoppers in Bangladesh

**DOI:** 10.1016/j.heliyon.2024.e35514

**Published:** 2024-07-31

**Authors:** Moumita Kar, S.M. Hemayet Jahan, Mohammad Atikur Rahman, Shuvo Dip Datta

**Affiliations:** a Department of Entomology, Patuakhali Science and Technology University, Dumki, 8602, Patuakhali, Bangladesh; b Department of Building Engineering and Construction Management, Khulna University of Engineering & Technology, Khulna, 9203, Bangladesh

**Keywords:** Rice, Brown planthopper, Insecticides, Molecular identification, Eco-friendly management

## Abstract

Infestation by various insect pests is the main constraint for growing rice where rice brown planthopper (*Nilaparvata lugens* Stål) can severely damage rice plants directly through feeding. Therefore, the study aims to detect rice brown planthoppers (BPH) and provide environment-friendly management tactics to mitigate the problem which caused by brown planthoppers. The BPH samples were collected from rice fields of different locations in the Patuakhali of Bangladesh for molecular identification. A molecularly single species of rice brown planthopper, *Nilaparavata lugens* was identified using mitochondrial cytochrome oxidase subunit I (mtCOI) universal marker. The nucleotide sequences of collected samples were compared with other nucleotide sequences from the GenBank database of NCBI, which make single clades in the phylogenetic tree at an insignificant distance. Moreover, brown planthopper management observations were recorded in laboratory conditions after providing an artificial diet with different treatments of plant-based insecticides Neem oil (1 %, 5 %, and 10 %), Castor oil (1 %, 5 %, and 10 %) where only 20 % sucrose solution was used as negative control and Abamectin (1 %, 5 % and 10 %) were also used as a positive control for comparing the efficacy of plant-based insecticides on rice brown planthoppers. The results showed the highest mortality (100 %) of rice brown planthoppers was recorded by Abamectin 10 %, followed by Abamectin 5 %. Neem 10 % performed better than Abamectin 1 % during 1st hour. Initial after exposure of 2nd hour for Abamectin 1 % revealed greater mortality (59 %) than Neem 10 %. Neem 5 % showed less effect on mortality in brown planthopper than Neem 10 % but was higher than Neem 1 % during 6 h of observation. The Castor oil of 10 % caused higher mortality than the Castor of 5 % but not up to the marks of Abamectin and different concentrations of Neem oil. Castor oil of 1 % and control have shown no mortality of brown planthopper for 6 h of observation.

## 1 Introduction

Bangladesh is a predominantly agricultural country. Rice dominates the cropping pattern across the country, with about 90 % of the people relying on it. Rice provides half of Bangladesh's agricultural GDP and one-sixth of the country's total income [1]. Rice covers approximately 74.35 percent of Bangladesh's total cropping area. However, many insect pest species find it to be a perfect host. Every year, farmers experience a variety of issues when it comes to farming rice due to severe infestations of various pests. According to Kumar et al. [2] and Liu et al. [3], the brown planthopper (BPH) is the most devastating pest in areas that cultivate rice, resulting in crop losses of thousands of dollars annually. Among the numerous pests associated with rice, brown planthoppers have been reported to infect rice in every region of the world that grows rice [4]. The BPH was first introduced in the southern region of Korea in 1912 and is now recognized as the country's most significant rice pest [5]. In Bangladesh, BPH was first officially documented in 1969. The first recorded outbreak of this insect was on Boro rice close to Dhaka city in April and May of 1976 [6]. When it came to transplanted aman rice, the main outbreak regions were found to be Rajshahi, Gazipur, Mymensingh, and Netrokona. Netrokona and Nandigram exhibited a high frequency of BPH, which resulted in a noticeable yield loss [7]. Like maize aphids, brown plant hoppers infest rice crops at every stage of plant growth [8]. In strong infestation, plants become yellow and quickly dry out, whereas mild infection reduces plant height, crop vigor, and tiller output. Early infestation begins as circular yellow spots that quickly become brownish as the plants dry up. A frequent term for feeding damage produced by BPH is “hopper-burn.” The infected areas have the potential to grow and cover the whole field. Moreover, it serves as a vector for the viral infections such as ragged stunt, grassy stunt and wilted stunt [9]. Improving the productivity of irrigated paddy fields is essential if rice production is to keep pace with population growth.

Nowadays, it is a great challenge to improve the rice field due to several pest attacks. Without precise identification, effective pest control of crop-damaging pest species is impossible. However, morphological identification of pests is sometimes challenging because of the significant physical similarities, noticeable colour variation, and smaller size variations during the nymph stage, whereas molecular techniques make the identification process very simple [10]. In Bangladesh, no one has yet used molecular tools to confirm them at the species level. Therefore, it's essential to identify each species of rice planthopper in order to effectively control them when they initially begin to spread over paddy fields. The main problem is that due to heavy infestation, farmers rely mainly on insecticides to control the high population densities of brown planthoppers in rice fields. However, indiscriminate use of highly toxic and persistent pesticides is producing a lot of issues, including disruption of natural enemy complexes, insecticide resistance, secondary pest outbreaks, pest resurgence, and pollution of the environment. This research focuses on how efficient biopesticides improve paddy growth and productivity after controlling pest and rice diseases. Moreover, plant-based pesticides are given more attention in this era of environmental concern because they are biodegradable and less destructive to the environment.

**Fig. 1:**
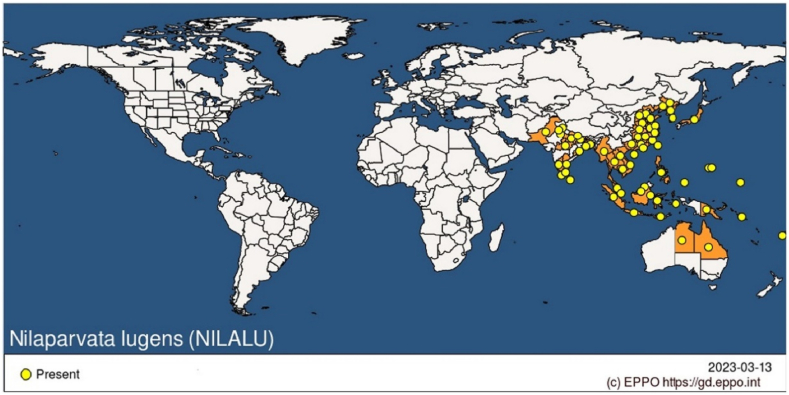
*Nilaparvata lugens*' worldwide distribution (Copyright: EPPO Global Database, obtained on March 13, 2023) [25].

In the case of the brown planthopper of Bangladesh, studies that are relevant to taxonomy and molecular research are almost nonexistent. However, information on molecular analysis and environment-friendly management of rice brown planthoppers is referred from nearby regions with the goal of reviewing and presenting the impact of various intervention strategies. In recent years, *Nilaparvata lugens* has seriously damaged rice crops in many tropical nations in the Orient and some Pacific islands [11]. It has also been observed in Sri Lanka [12], Vietnam [13], China [14], Bangladesh [15], India [16], Fiji [17], Korea [18], Japan [19], Papua New Guinea [20], Indonesia [21], Papua New Guinea [20], Solomon Islands [22], Thailand [23], and Malaysia [24] as presented in Fig. 1.

The genus *Nilaparvata* is believed to contain 14 known species and two unknown species worldwide. They are *N. angolensis Synave, N. caldwelli Metcalf, N. bakeri (Muir), N. chaeremon Fennah, N. camilla Fennah, N. albotristriata (Kirkaldy), N. diophantus Fennah, N. maeander Fennah*, *N. myersi Muir, N. muiri China, N. seminula Melichar, N. nigritarsis Muir, N. lugens (Stål.), N. wolcotti Muir et Giffard, N. sp. a, N. sp. b.* The most crucial feature that sets the species apart is its genitalia, particularly the styles (parameters) and aedeagi for males and lateral lobes (1st valvifers) for female adults [26]. In order to successfully identify the insects at the species level, a number of studies have used molecular techniques, such as restriction fragment length polymorphism-based conventional polymerase chain reaction (PCR) [27,28], multiplex PCR [28], loop-mediated isothermal amplification (LAMP) assays [29,30] which are not restricted to the growing or adult phases of the target planthopper types. Only one species may be identified at a time using conventional PCR, whereas multiplex PCR has the ability to detect more than two species at a time. In the absence of genomic DNA (gDNA) isolation, the LAMP test can be helpful for field diagnosis [31]. Due to sequence similarities of different species and the sensitivity of the PCR conditions, it is important to create suitable primers and optimize the reaction conditions to successfully identify the insects at the species level [32]. Mitochondrial DNA (mtDNA) provides a suitable marker because of its simple genetic structure, low rate of recombination, maternal inheritance, and relatively quick rates of evolution [33,34]. However, molecular identification facilitates the process of controlling pest populations by adopting appropriate management practices.

For the eco-friendly management of insects, Ramli et al. [35] investigated the impact of using a combination of natural botanical extracts and a neem-derived biopesticide for crop protection during the early stages of paddy cultivation. The study compared three conditions: T1 (paddy sprayed with both BV500WS and BV612EC biopesticides), T2 (paddy treated solely with BV500WS biopesticide), and C1 (control with no pesticide use). The research involved releasing BPH twice during the cultivation process. Results demonstrated that T1 achieved the highest percentage of remaining tillers (68.56 %) and a significant reduction in the BPH population with a mortality rate of up to 100 %. In contrast, T2 maintained a similar tiller percentage to C1, despite BPH impact, with tiller percentages of 46.24 % and 49.65 % respectively. The study concluded that using two biopesticides is more effective than a single type in controlling BPH levels. Another research study has been done on a botanical insecticide that combines ginger and chili extracts [36]. Three factors—the concentration of the extract, the exposure period, and the exposure temperature—were examined. The findings demonstrated that 90 % of BPH deaths occurred at 40 % extract concentration after 72 h of exposure at the ideal temperature of 30 °C. The presence of gingerol and capsaicin was demonstrated by HPLC analysis, with peaks occurring at 4.502 and 11.046 min, respectively. The BPH demonstrated preferential repel action against the treated paddy based on repellency analysis. Using various solvents and additives, Rismayani et al. [37] investigated the effectiveness of botanical insecticide formulas with eugenol derived from Clove oil. It focuses on comparing the toxicity of 12 different botanical insecticide formulas against *N. lugens* adults by applying the solutions to rice plants. The experiment determines the most effective formula, consisting of 500 ml of clove oil, 450 ml of kerosene, and 50 ml of Silwet HS 312, which also proves to be cost-efficient. The study findings of Hernani et al. [38] indicated that seven different formulations of natural pesticides based on liquid rice hull smoke are highly effective in controlling brown planthopper, with mortality rates exceeding 90 % at a 10 % concentration over seven days. The study also reports the toxicity levels of these formulations, with LD50 values ranging from 128 to 725 ppm, categorizing them as toxic and suitable for use as pesticides. Telaumbanua et al. [39] examined the impact of pests such as stinky bugs, brown planthoppers, and others on rice production, emphasizing both the effectiveness of chemical pesticides and their ecological drawbacks. The study evaluates the application of citronella-based pesticides, demonstrating notable reductions in pest populations in treated plots. However, it also highlighted a concurrent decline in predatory insect numbers, suggesting the necessity of integrated pest management strategies to maintain ecological balance in agricultural settings.

From the above literature, it was observed that there is a lack of studies on molecular identification and management of brown planthoppers in perspective of Bangladesh. Bear in mind the concept of the agro-ecosystem of the country, the purpose of the study was to identify the species of brown planthoppers and to determine how effectively various bio-pesticides worked against it. In order to identify the species of collected BPH, the authors have employed molecular techniques using mitochondrial cytochrome oxidase subunit I (mtCOI) marker and compared the sequences with other nucleotide sequences from the GenBank database of NCBI. For assessing the management of BPH, the sandwich technique has been employed, providing an artificial diet. Moreover, observations on brown planthopper management were recorded in laboratory conditions using various concentrations of plant-based insecticides (Neem oil and Castor oil), and Abamectin was used as a positive control for comparing the efficacy of plant-based insecticides on rice brown planthoppers.

## 2 Materials and methods

This research was carried out from April 2021 to February 2022 in the Insect Molecular Physiology Laboratory of Kyungpook National University in Korea for the molecular identification and eco-friendly management of rice brown planthoppers based on bio-assay with insecticides in the Entomology lab of Patuakhali Science and Technology University.

### 2.1 Site selection

**Fig. 2:**
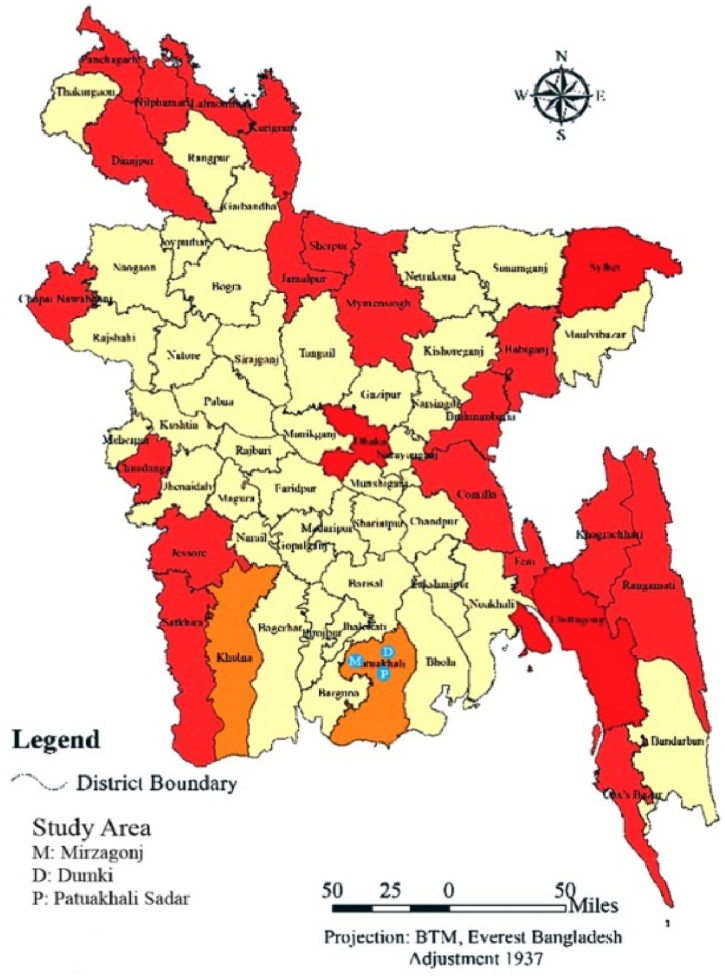
Map of Bangladesh.

**Fig. 3:**
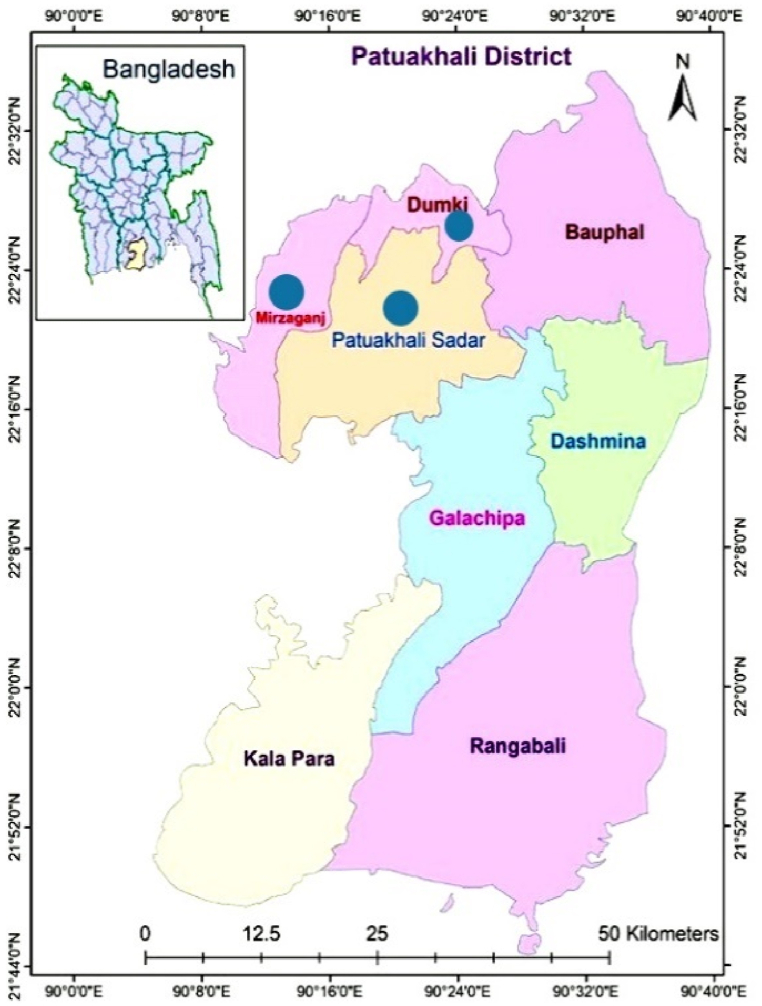
Map of Patuakhali district.

Geographically, the experimental site is located at latitude 22.3542 °N and longitude 903181°E in Patuakhali district, Bangladesh, as presented in Fig. 2, Fig. 3. Rivers encircle the district on three sides. It is located in the division of Barishal. The Bay of Bengal belongs to this district. The districts cover 3220.15 km^2^ of land.

### 2.2 Crop selection

Rice fields were selected from the Patuakhali region of Bangladesh. Planthopper samples were collected from infested rice plants of selected fields.

### 2.3 Climate

The Patuakhali region of Bangladesh has tropical weather. In Patuakhali, there is heavy rainfall throughout the majority of the year. Rainfall in winter is significantly lower than in summer. 25.9 °C is the typical annual temperature and 2654 mm of rain falls on average each year. The difference in rainfall between the wettest and driest months is 576 mm. The average temperature varies by 10.6 °C throughout the year**.**

### 2.4 Molecular analysis

#### 2.4.1 Sample collection

**Fig. 4:**
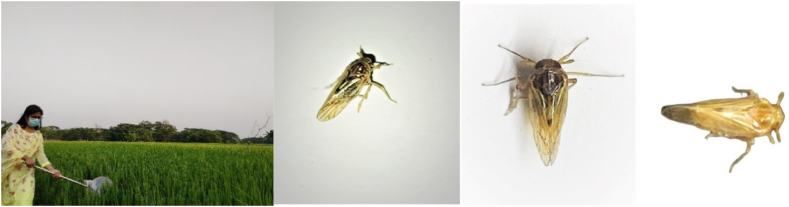
Collected samples of rice brown planthoppers.

Rice brown planthoppers were collected by a sweeping net (30 cm diameter), from the rice field of the Patuakhali district of Bangladesh. Fig. 4 shows the collection process and some collected samples of brown planthoppers. The planthoppers were collected by 10 sweeps from each plot of different places, and counted separately which were kept in a plastic bag for further analysis. Table 1 presents the number of brown planthoppers collected from different regions of Patuakhali. Collected specimens (nymph and adult) of both sexes of brown planthoppers were preserved separately in absolute ethanol and kept at −20 °C until DNA extraction.

**Table 1:** Collection chart of Rice Brown planthopper.

Sample no.	Species	Location	No. of collected sample
1.	*Nilaparavata lugens*	Patuakhali	130
2.	*Nilaparavata lugens*	Patuakhali	145
3.	*Nilaparavata lugens*	Patuakhali	186
4.	*Nilaparavata lugens*	Patuakhali	210
5.	*Nilaparavata lugens*	Patuakhali	166

#### 2.4.2 DNA extraction

To prevent cross-contamination between species, genomic DNA was isolated from single adult individuals using a PureLink Genomic DNA Mini Kit (Invitrogen, Carlsbad, CA, USA), with minor adjustments to the manufacturer's instructions. One planthopper was placed in a 1.5 ml Eppendorf tube with 20 μl of proteinase K (50 μg/ml) and 200 μl of digestion buffer, and it was left to sit at 55 °C for 12 h. Centrifugation at 10,000 rpm for 1 min was used to wash the mixture through the genomic spin column after it had been combined with the genomic lysis/binding solution (200 μl) and 100 % ethanol (200 μl). Following two rounds of repeated washings with the Wash buffer, DNA was eluted using the Elution buffer (20 μl) and centrifuged for 1 min at 12000 rpm into a fresh E-tube. The concentrations of DNA samples that had been purified were measured with an Implement Nano spectrophotometer located in Schatzbogen, Germany.

#### 2.4.3 PCR amplification

Amplifying mitochondrial cytochrome oxidase subunit 1 (mtCOI) regions was carried out from the DNA of collected samples using universal primer sets of mtCOI. The primer set for mtCOI region is as follows: forward (LCO1490) (5′- GGTCAACAAATCATAAAGATATTGG -3′) and reverse (HCO2198) (5′- TAAACTTCAGGGTGACCAAAAAATCA -3′) primers which amplified the central region of COI [40]. PCR was performed in 25 μl of Smart Taq Pre-Mix (Solgent, Daejeon, Korea), comprehending 40 ng of DNA as a template and 10 pmol of each primer (Table 2). The following parameters were used for boosting up the mixtures: a 5-min initial denaturation at 95 °C, thirty-five cycles of 60 s at 95 °C, 60 s at 40 °C, and 90 s at 72 °C, and a 7-min final elongation step at 72 °C. PCR results were examined using 1 % agarose gel electrophoresis after staining with ethidium bromide solution and exposed under UV light for visualization (Table 3).

**Table 2:** Primer information for brown planthopper identification.

Use for Detection	Primer Name	Primer Direction	Primer Sequence (5′ to 3′)	Size(bp)	Reference	Annealing Temp.
BPH	LCO1490 HCO2198	Forward Reverse	GGTCAACAAATCATAAAGATATTGG TAAACTTCAGGGTGACCAAAAAATCA	710	Folmer et al., 1994	40 °C 35 cycles

**Table 3:** PCR condition for brown planthopper (BPH) identification.

Use	Targeted Gene	Pre-denaturation	Denaturation	Cycling conditions		
				Annealing	Extension	Cycles
BPH	mtCOI	95 °C (5 min)	95 °C (60 s)	40 °C (60 s)	72 °C (90 s)	35

#### 2.4.4 DNA sequencing and phylogenetic analysis

The PCR products were sequenced in an Applied Biosystems 3100 Capillary DNA Sequencer (Solgent, Daejeon, Korea) using a BigDye Terminator Cycle Ordering Kit (Applied Biosystems, Foster City, CA, USA). The PCR products were separated on a 2 % LMP agarose gel, excised, and purified using the Wizard PCR preps DNA purification system (Promega, Madison, WI, USA). The sequences that were obtained were aligned using Clustal Omega [41] alignment software and compared to previously deposited sequences in NCBI databases [42]. Molecular evolutionary genetic analysis (MEGA) was used to assess sequence divergence among taxa based on Kimura-2-parameter (K2P) distances [43]. Utilizing the neighbor-joining technique [44] and MEGA Software Version 6.0 [45], phylogenetic analyses were carried out. From 1000 repeats, bootstrap values were derived [46].

#### 2.4.5 Alignment and characterization of gene fragments

BioEdit 7.0.5.3 will be used to review and make corrections to raw sequences [47]. Clustal Omega was used to align the sequences of the COI genes [48]. The MEGA 6.0 program was used to extract base frequencies, pairwise sequence divergences, number of substitutions, and transition/transversion ratios [49].

#### 2.4.6 Experimental treatments

The following treatments were used for ecofriendly management of brown planthoppers:

To: Only 20 % sucrose solution was used as a negative control.

T_1_: Abamectin 1 % was used as the positive control.

T_2_: Abamectin 5 % was used as the positive control.

T_3_: Abamectin 10 % used as the positive control.

T_4_: Neem oil 1 %

T_5_: Neem oil 5 %

T_6_: Neem oil 10 %

T_7_: Castor oil 1 %

T_8_: Castor oil 5 %

T_9_: Castor oil 10 %

The experiment was carried out in Randomized Block Design (RBD) with eight treatments. All the treatments were replicated three times simultaneously. It contains both biological and technical replicates.

#### 2.4.7 Preparation of botanical extraction and artificial diet

The abamectin 1.8 EC, neem extract oil, and castor extract oil were purchased from the nearby market. The 20 % sucrose solution was prepared in a beaker, and 1 %, 5 % and 10 % of abamectin solution were prepared separately in other beakers. Then, an artificial diet for BPH was prepared by 1:1 ratio using sucrose solution with Abamectin. The other extracting agents, i.e., Neem extract oil and Castor oil, followed the same procedure for the preparation of an artificial diet. While effective as insecticides, plant oils can adversely affect plant physiology if applied in high concentrations [50]. Plants rely on various physiological processes, including photosynthesis, which can be disrupted by excessive oil concentration on their surfaces. Research indicates that plants typically cannot tolerate oil concentrations exceeding 15 % without experiencing detrimental effects on their growth and development. Therefore, in this study, concentrations ranging from 1 % to 10 % were chosen to ensure that the oil substances did not exceed levels that could compromise plant health. Unlike synthetic pesticides, which may have longer residual activity, natural insecticides derived from plant oils tend to be short-lived on plants. Consequently, they may provide temporary protection against pest invasions and require more frequent applications to maintain efficacy.

### 2.5 Mortality observation by parafilm assay

In this study, the bio-assay experiment was performed in the lab to confirm the toxicity of plant-based insecticides due to the difficulties of counting mortality in field situations. Moreover, this study used two controls: a chemical pesticide, Abamectin used as a positive control and a 20 % sucrose solution used as a negative control or neutral for comparing the efficacy of plant-based insecticides like neem oil and castor oil. Abamectin is used here as a marker of whether the experiment is working or not.

**Fig. 5:**
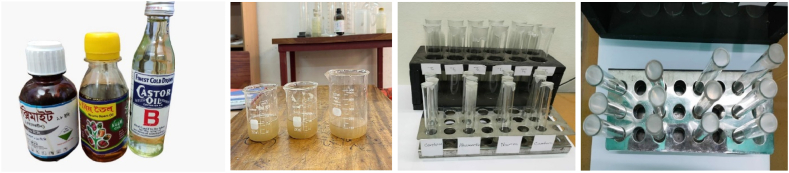
Preparation of artificial diet and assessment of mortality percentage.

A 20 % sucrose solution artificial diet was made using distilled water and stored at 4 °C until it was needed. The brown planthoppers were kept in feeding chambers made of glass tubes measuring L-20 cm × Ø 3 cm. A double-layered sandwich of parafilm with a 20 % sucrose solution sandwiched in between was placed over the upper end of the chamber. An artificial diet solution (50 μl containing 20 % sucrose) was put in the parafilm sandwich. A lid with a hole in the middle covered the bottom end of the chamber. Using a pipette tip and a fine-meshed net glued onto the bottom end, a makeshift ventilator was able to close the hole. Ten adult brown planthoppers were placed in a glass chamber and allowed to suction the artificial diet solution through the parafilm. For up to 6 h, dead insects were counted every 30 min. Every experiment set was run three times under the identical circumstances. A similar experimental method was obtained in the study of Yasudomi et al. [51]. Fig. 5 shows the artificial diet preparation process for assessing BPH mortality in lab conditions.

### 2.6 Meteorological data

Simultaneously, meteorological data such as maximum and minimum temperature, rainfall, relative humidity, and wind velocity were collected from the meteorological center at Patuakhali, Bangladesh.

### 2.7 Statistical analysis

Each data set of rice brown planthopper was represented as three separate replicates, and Sigma plot 8.0 (Systat Software, Inc., Point Richmond, CA, USA) was used to plot the mean values with standard error. Using the PROC General Linear Model (GLM) and the Statistical Analysis System (SAS, 2002–2003 SAS Institute Inc., Cary, NC, USA) version 9.1 software, analysis of variance (ANOVA) was used to determine means. Duncan's Multiple Range Tests were used to identify significant variations between mean values (DMRT). Three separate replicates were used to assess the data in a randomized design. Sigma Plot 8 was employed for the graphical depiction.

## 3 Results

### 3.1 Molecular identification of rice Brown planthoppers

**Fig. 6:**
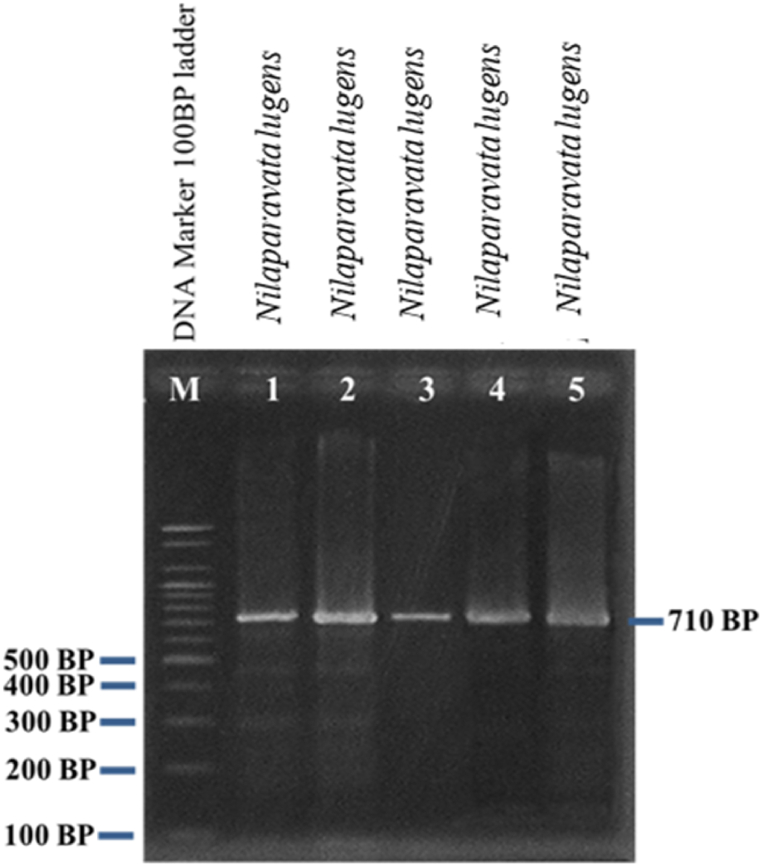
Brown planthoppers detection based on a fragment (∼710 bp) of the amplified mtCOI DNA using 2 % agarose gel electrophoresis and visualized by UV light after staining with ethidium bromide.

Rice brown planthoppers were collected from different places in the Patuakhali district and belonged to the species *Nilaparavata* sp*.* It has been shown to the band at 710bp to LCO1490(F) and HCO2198(R) primer. The rice brown planthopper *Nilaparavata lugens* was identified from collected specimens using Polymerase Chain Reaction and the comparison of the mtCO1 gene from the NCBI database (Fig. 6).

### 3.2 mtCOI sequences analysis of rice Brown planthoppers

The length of obtained PCR products of the mtCOI was approximately 710 bp in all examined samples (Fig. 8). In search of determining sequences in the GenBank database from the National Centre for Biotechnology Information (NCBI), species were identified by 100 % identical values of mtCOI sequences. The sequences of the collected samples no. 1, 2, 3, 4, and 5 all showed 100 % similarity with the species of *Nilaparavata lugens.*

### 3.3 Phylogenetic analysis

**Fig. 7:**
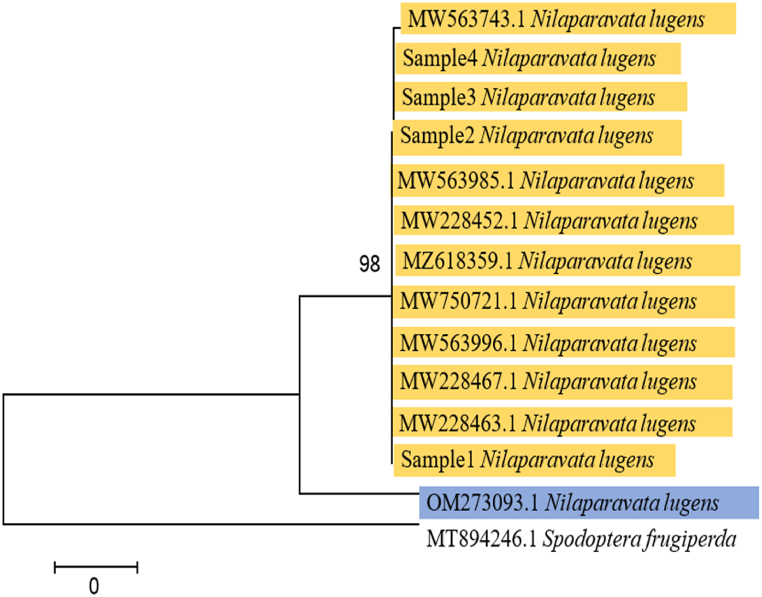
A phylogenetic tree is constructed from the mitochondrial COI sequences of different brown rice planthopper species using the neighbor-joining (NJ) method. The values next to branches are the bootstrap values (more than 50 %) from 500 trials. The bar shows the distance in terms of phylogeny.

Phylogenetic trees were constructed by the neighbor-joining (NJ) method using mtCOI sequences of single species of *Nilaparavata lugens* in the family Delphacidae, which were identified in this study using the deposited sequence data from the NCBI GenBank database. COI sequences of *Spodoptera frugiperda* which was used as outer groups of this cladogram. The results showed that four mtCOI sequences of brown planthoppers, which were collected from rice fields in the Patuakhali region of Bangladesh, were clustered in single clades with MW563743 in phylogenetic trees (Fig. 7). The results showed that phylogenetic trees of mtCOI were strongly clustered within the same species.

**Fig. 8:**
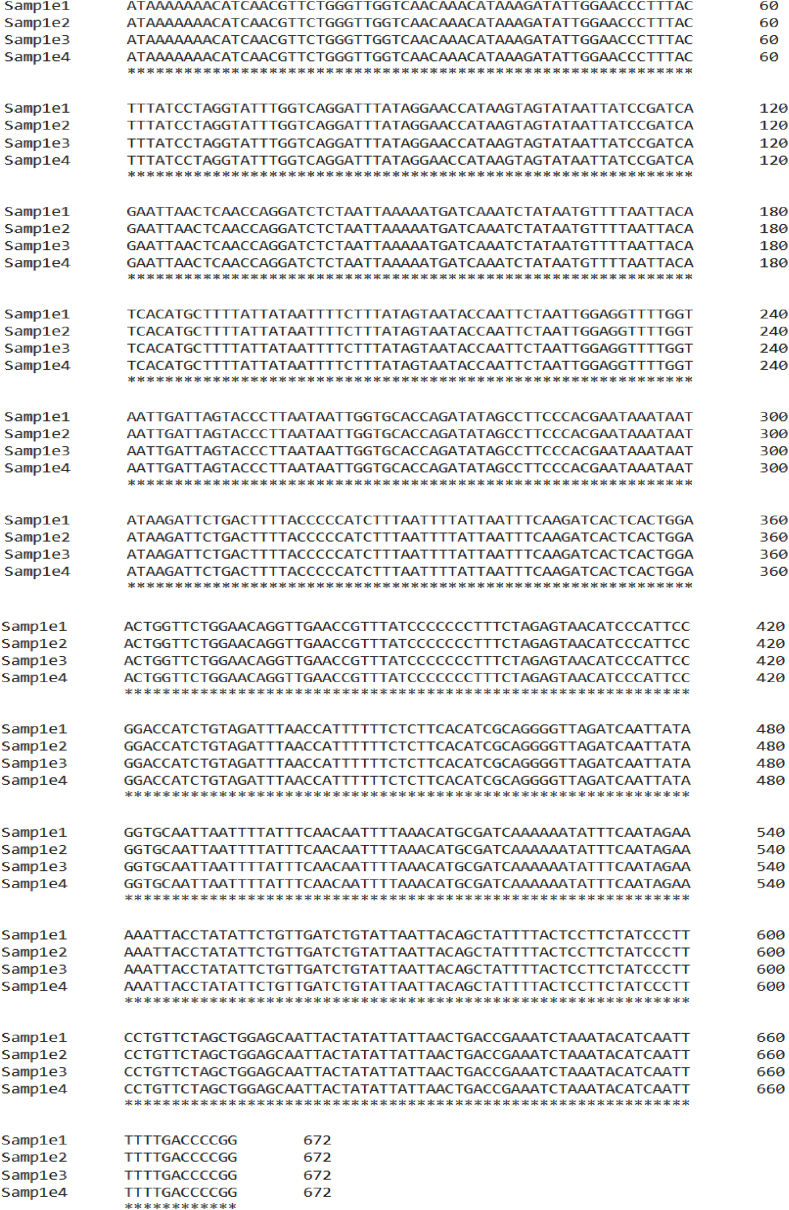
Sequence alignment of collected planthoppers samples using the Clustal Omega program for ecofriendly management of BPH.

### 3.4 Molecular distance analysis using nucleotide frequency and composition

The nucleotide composition of mtCO1 sequences for single species of brown planthopper was analyzed by Mega 4.0 and found all nucleotide frequency ratios of T: C: A: G were 37.5, 16.3, 32.2, 14.0 percent, and the ratio of nucleotide composition at conserved site of T: C: A: G were also 37.5, 16.3, 32,2, 14.0 percent for *Nilaparavata lugens*. The ratio of variable nucleotide composition of T: C: A: G was 33.5, 20.3, 26.5, and 19.7, as shown in Table 4.

**Table 4:** Nucleotide frequency and composition in different site.

	All nucleotide Frequency (%) 606/606					Conserved Nucleotide (%) 514/635					Variable Nucleotide (%) 92/635				
BPH Species	**T(U)**	**C**	**A**	**G**	**Total**	**T(U)**	**C**	**A**	**G**	**Total**	**T(U)**	**C**	**A**	**G**	**Total**
*N. lugens*	37.5	16.3	32.2	14.0	680	37.5	16.3	32.2	14.0	680	33.5	20.3	26.5	19.7	325
*N. lugens*	37.5	16.3	32.2	14.0	680	37.5	16.3	32.2	14.0	680	33.5	20.3	26.5	19.7	325
*N. lugens*	35.9	18.9	32.4	12.8	672	35.9	18.9	32.4	12.8	672	30.5	24.9	27.1	17.5	325
*N. lugens*	37.6	16.5	32.1	13.8	680	37.6	16.5	32.1	13.8	680	33.8	20.6	26.2	19.4	325
**Average**	**37.1**	**17.0**	**32.2**	**13.6**	**678**	**37.1**	**17.0**	**32.2**	**13.6**	**678**	**32.8**	**21.5**	**26.5**	**19.1**	**325**

### 3.5 Sequence alignment of collected planthoppers

Rice brown planthopper sequence alignment was done by the Clustal Omega Program. This sequence alignment clearly identifies the nucleotide similarity between the samples molecularly identified from Patuakhali. The sequence alignment of collected planthoppers is presented in Fig. 8.

#### 3.5.1 Result of mortality using artificial diet

**Fig. 9:**
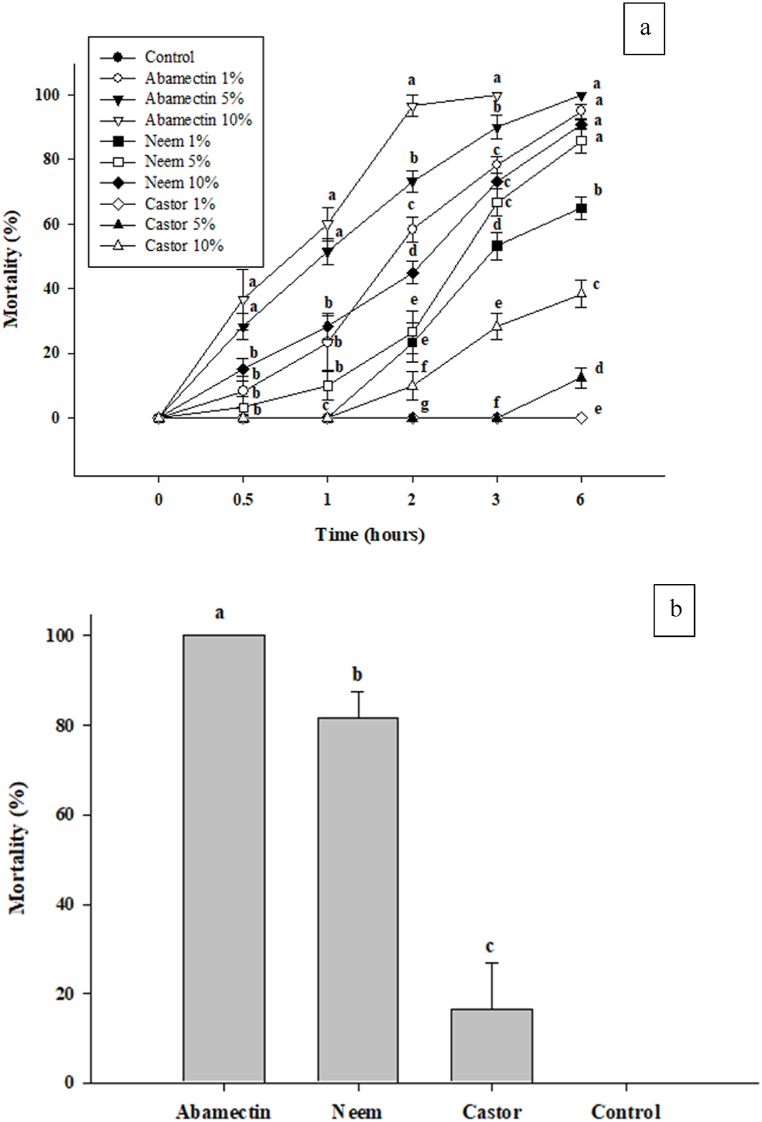
(a) The mortality percentage with time; (b) Mortality percentage variation of rice brown planthopper.

As a part of the management, rice brown planthoppers were treated by following management treatments with three replications i.e. Control (T_0_), Abamectin 1 % (T_1_), Abamectin 5 % (T_2_), Abamectin 10 % (T_3_), Neem oil 1 % (T_4_), Neem oil 5 % (T_5_), Neem oil 10 % (T_6_), Castor oil 1 % (T_7_), Castor oil 5 % (T_8_), Castor oil 10 % (T_9_). In Fig. 9, it was observed that the highest mortality of rice brown planthoppers was recorded by Abamectin 10 % followed by Abamectin 5 %. Neem 10 % worked more than Abamectin 1 % for up to 1 h. After 1 h, Abamectin 1 % causes greater mortality than Neem 10 %. Neem 5 % causes less mortality of brown planthopper than Neem 10 % but greater than Neem 1 % up to 6 h. Castor 10 % causes greater mortality than Castor 5 % but worked less than Abamectin and Neem concentration. Castor 1 % and control causes no mortality of brown planthopper up to 6 h.

Fig. 9 showed that the highest mortality percentage of rice brown planthopper was recorded by Abamectin, followed by Neem, as compared to castor oil and control. Abamectin causes 100 % of mortality in brown planthoppers, whereas Neem causes 80 % of deaths. Less than 20 % of mortality was observed by applying castor oil. No death of insects was observed in control.

## 4 Discussion

Using molecular techniques, the study successfully identified the rice brown planthopper species *Nilaparvata lugens* from samples collected in the Patuakhali district, Bangladesh. The identification was confirmed through PCR targeting the mtCOI gene, with a consistent amplification product of approximately 710 base pairs across all samples. This approach utilized the primers LCO1490(F) and HCO2198(R), which are well-established for barcoding in insect species. The results were validated by comparing the amplified sequences to the NCBI GenBank database, revealing a 100 % match to *N. lugens*. The accuracy and reliability of using the mtCOI gene for species identification were thus confirmed, aligning with previous studies that have demonstrated the utility of mtCOI barcoding in defining insect species [10].

Employing the NJ method, the phylogenetic analysis clustered the mtCOI sequences from the collected planthoppers into a single clade with a reference sequence (MW563743) from the GenBank database. This strong clustering within a single clade underscores the genetic homogeneity of the *N. lugens* populations in the particular region. The use of *Spodoptera frugiperda* as an outgroup further validated the specificity and distinctiveness of the mtCOI sequences of *N. lugens*. These findings provide insight into the genetic structure and potential gene flow among brown planthopper populations, indicating a lack of significant genetic divergence within the studied area. This is consistent with similar studies conducted in other regions, where mtCOI-based phylogenetic analyses have also shown high genetic similarity among geographically proximate populations of *N. lugens* [52].

The analysis of nucleotide frequency and composition revealed a consistent pattern across all samples. The conserved sites exhibited a nucleotide frequency of T: C: A: G at 37.5 %, 16.3 %, 32.2 %, and 14.0 %, respectively. This uniformity in conserved nucleotide composition suggests strong evolutionary pressures maintaining sequence stability within the species. However, the variable nucleotide sites displayed different frequencies (T: 33.5 %, C: 20.3 %, A: 26.5 %, G: 19.7 %), reflecting regions of the genome more susceptible to mutation and potential adaptation. These findings highlight the genetic variability within *N. lugens*, which could be critical for understanding mechanisms of adaptation and resistance to environmental pressures, including insecticides.

The sequence alignment performed using the Clustal Omega program provided a detailed comparison of nucleotide similarities among the collected samples. The high degree of sequence alignment among the samples confirms the species identification and suggests limited genetic variation within the *N. lugens* population in the study area. This alignment also aids in the detection of any potential polymorphisms or mutations that could be relevant for pest management strategies. Compared to other findings, similar patterns of conserved and variable site compositions have been observed in related studies, reinforcing the generalizability of these results across different populations and geographic locations [53].

Farmers mostly depend on pesticides to manage their crops, and over 50 % of all insecticides used on rice are targeted, especially this pest [54]. Because of this, the most frequent technique for managing BPH in Bangladesh is the application of chemicals. Even though many commonly used pesticides have been tested against this pest, most of the chemicals have not been able to control it effectively. However, excessive use of chemical pesticides can lead to several issues, such as the emergence of biotypes resistant to the insecticide, contamination of the environment, and negative impacts on species other than the target, such as the target's natural enemies and an epidemic of secondary pests [55]. The farmers rarely wear the required personal protection equipment, smoke while at work, don't wash their hands carefully, and dispose of the packaging incorrectly. Botanical pesticides can reduce this problem. In the Indonesian Spices and Medicinal Crops study Institute, a screen house study was carried out by testing the botanical pesticide formulation at five different concentrations (0.0, 0.5, 2.5, 4.5, and 6.5 cc/l), and doing so five times [56]. The outcomes demonstrated that the pesticide could kill 97 % of the insects at a dosage of 4.5 cc/l. Additionally, research shows that this particular pesticide is readily biodegradable in the environment, means that it leaves no residue on agricultural products. Furthermore, the active components only affect specific plants and do not contaminate water, soil, air, or other natural resources. Nor do they create insect immunity or resistance [56]. Therefore, several biopesticides are being assessed in an effort to minimize the disruption of the environment's quality. The results of the mortality assays underscore the effectiveness of different treatment concentrations of Abamectin, neem oil, and castor oil in managing *N. lugens* populations. Abamectin at a 10 % concentration demonstrated the highest efficacy, achieving 100 % mortality within 3 h, significantly outperforming lower concentrations and other treatments. Neem oil at 10 % also showed substantial efficacy, with 80 % mortality observed, indicating its potential as a bio-pesticide. The comparison between abamectin and neem oil suggests that while Abamectin is more compelling, neem oil offers a safer, environmentally friendly alternative.

The differential mortality rates observed with neem oil at varying concentrations highlight its dose-dependent effectiveness, with higher concentrations providing more rapid and higher mortality. Although less effective than Neem and Abamectin, Castor oil still showed some potential, particularly at higher concentrations. These findings support the use of neem oil as a viable bio-pesticide, especially in integrated pest management programs where reducing chemical pesticide use is a priority. When compared to other studies, the efficacy of neem oil, as observed in this study, aligns well with findings in other regions, where neem oil has been shown to effectively manage a variety of agricultural pests, including *N. lugens* [57]. Similarly, the high mortality rates caused by Abamectin verify previous research that highlights its effectiveness against resistant pest populations [58].

## 5 Implications of the study

The study's findings have significant implications for the development of integrated pest management (IPM) strategies for *N. lugens* in rice cultivation. The molecular identification and phylogenetic analysis provide a robust foundation for monitoring and managing pest populations. The demonstrated effectiveness of neem oil as a bio-pesticide offers a sustainable alternative to chemical pesticides like Abamectin, potentially reducing environmental impact and promoting ecological balance.

The genetic insights into *N. lugens* populations, coupled with the efficacy data of various treatments, enable the design of targeted and efficient pest management protocols. Future research could focus on understanding the mechanisms of resistance development in *N. lugens* to various pesticides and exploring the synergistic effects of combining botanical and chemical treatments. This holistic approach could enhance the sustainability and resilience of rice production systems against brown planthopper infestations.

Overall, the study provides comprehensive insights into the molecular identification, genetic characterization, and effective management of rice brown planthopper populations in the Patuakhali district. The integration of molecular techniques with practical pest management solutions underscores the potential for innovative and sustainable approaches to crop protection.

## 6 Conclusion

Considering the assessment's findings, a molecularly single species of rice brown planthopper *Nilaparavata lugens* was identified from the Patuakhali region of Bangladesh by the nucleotide sequences in comparison. The mtCO1 sequence of collected rice brown planthopper makes single clades in the phylogenetic tree with insignificant distance. Comparing the performance of different concentrations of the three pesticides for controlling the brown plant hopper, *N*. *lugens,* it was observed that the chemical pesticide Abamectin 10 % caused 100 % mortality within 3 h, whereas the botanical pesticide Neem 10 % caused almost 80 % mortality in the same time. Along with that, in the first hour, Neem worked 10 % more than Abamectin 1 %. Though a higher concentration of Abamectin works faster than Neem, being a chemical pesticide, it is not always safe for the environment. Neem is safe as it is a botanical biocide that acts on targeted insect pests. So, if it is considered the time frame, Neem can be an effective bio-pesticide for controlling rice brown planthopper.

Further studies will be conducted on experiments over longer periods to capture data on the persistence of insecticidal activity. Moreover, employing advanced analytical techniques to measure insecticide residues in plant tissues and the environment can add additional dimensions. Furthermore, the impact on non-target organisms and ecosystem health should be evaluated through ecotoxicological assessments in the future.

## Funding

This work was supported by the 10.13039/501100008804Ministry of Science and Technology of Bangladesh through the National Science and Technology (NST) Fellowship Program for the development of science and technology.

## Data availability

The datasets generated during and/or analyzed during the current study are available from the corresponding author on reasonable request. All data generated or analyzed during this study are included in this published article done by the author.

## Consent for publication

All participants gave their consent for their data to be published in the journal article.

## CRediT authorship contribution statement

**Moumita Kar:** Writing – review & editing, Writing – original draft, Visualization, Validation, Software, Resources, Methodology, Investigation, Formal analysis, Data curation, Conceptualization. **S.M. Hemayet Jahan:** Visualization, Validation, Supervision, Resources, Project administration, Methodology, Funding acquisition, Formal analysis, Conceptualization. **Mohammad Atikur Rahman:** Writing – review & editing, Validation, Supervision, Project administration, Funding acquisition, Formal analysis, Conceptualization. **Shuvo Dip Datta:** Writing – review & editing, Writing – original draft, Visualization, Validation, Resources, Data curation.

## Declaration of competing interest

The authors declare the following financial interests/personal relationships which may be considered as potential competing interests:Moumita Kar reports financial support was provided by the Government of the 10.13039/501100008804People's Republic of Bangladesh Ministry of Science and Technology. If there are other authors, they declare that they have no known competing financial interests or personal relationships that could have appeared to influence the work reported in this paper.
